# Cold Shock as a Screen for Genes Involved in Cold Acclimatization in *Neurospora crassa*

**DOI:** 10.1534/g3.118.200112

**Published:** 2018-03-21

**Authors:** Michael K. Watters, Victor Manzanilla, Holly Howell, Alexander Mehreteab, Erik Rose, Nicole Walters, Nicholas Seitz, Jacob Nava, Sienna Kekelik, Laura Knuth, Brianna Scivinsky

**Affiliations:** Department of Biology, Valparaiso University, Valparaiso, Indiana 46383

**Keywords:** *Neurospora*, cold shock, cold adaptation, morphology, branching, Mutant Screen Report

## Abstract

When subjected to rapid drops of temperature (cold shock), *Neurospora* responds with a temporary shift in its morphology. This report is the first to examine this response genetically. We report here the results of a screen of selected mutants from the *Neurospora* knockout library for alterations in their morphological response to cold shock. Three groups of knockouts were selected to be subject to this screen: genes previously suspected to be involved in hyphal development as well as knockouts resulting in morphological changes; transcription factors; and genes homologous to *E. coli* genes known to alter their expression in response to cold shock. A total of 344 knockout strains were subjected to cold shock. Of those, 118 strains were identified with altered responses. We report here the cold shock morphologies and GO categorizations of strains subjected to this screen. Of strains with knockouts in genes associated with hyphal growth or morphology, 33 of 131 tested (25%) showed an altered response to cold shock. Of strains with knockouts in transcription factor genes, 30 of 145 (20%) showed an altered response to cold shock. Of strains with knockouts in genes homologous to *E. coli* genes which display altered levels of transcription in response to cold shock, a total of 55 of 68 tested (81%) showed an altered cold shock response. This suggests that the response to cold shock in these two organisms is largely shared in common.

The environmental conditions that life must contend with can vary widely. Organisms have evolved a wide range of mechanisms for contending with these changing conditions. For the filamentous fungus *Neurospora*, growth continues through nearly the entire range of temperatures (above freezing) that is observed in this environment. Although the rate of tip extension varies linearly with temperature (Watters *et al.* 2000), the branch density (the statistical distribution of distances between branch sites along a linear growing hypha) remains constant across this range (Watters *et al.* 2000) allowing the fungus to continue to infiltrate its environment at the same density. Temperatures progressing through this range would be expected to have dramatic impacts on enzyme activity generally (and thus overall metabolism), but also directly on features critical to growth such as membrane fluidity, DNA/RNA stability and the rates of transcription and translation.

In both *Neurospora* and *E. coli*, there is a multistage response to cold shock. There is an initial response which is transient in nature, followed by a more long-term response which largely represents a return to normal growth. *Neurospora* grows via extension at a hyphal tip with periodic branching which is typically lateral (Figure 1A). However, when *Neurospora* is subjected to cold shock, a multi-phase morphological response is observed (Figure 1B, Watters *et al.* 2000, Watters 2013). The initial response to cold shock is the growth of a single longer than normal unbranched segment. This was termed the “Lag” phase of the response. This phase is followed by a series of closely spaced apical branch points, termed the “Apical” phase. Apical branch formation has been previously associated with the disruption and attempted reorganization of the normal tip-growth apparatus (Reynaga-Peña *et al.* 1995, Riquelme & Bartnicki-Garcia 2004), a mechanism distinct from that thought to be involved in lateral branching. Finally, with continued incubation at the lower temperature, the colony returns to lateral branching, termed the “Recovery” phase. Growth in this phase of the response resembles that which would be seen had the colony been grown at 4° (or any other fixed temperature) continuously (Watters *et al.* 2000). Thus, the cold shock response appears to be a temporary disturbance to a homeostatic system which maintains branch density at a constant, evolutionarily favored, value. The morphological effects of cold shock are the indirect consequence of this system’s staged process of adjusting cellular conditions in order to compensate for the new growth temperature.

**Figure 1:**
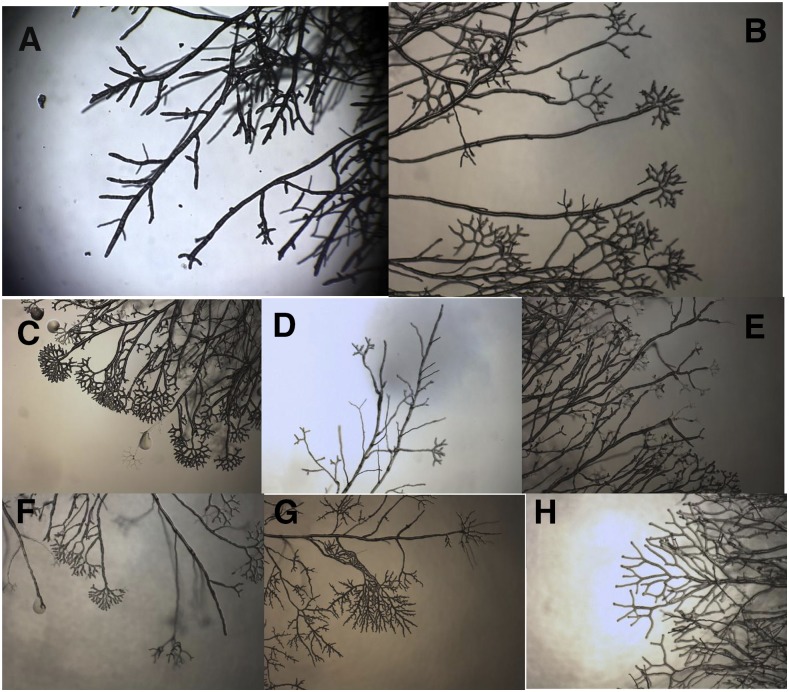
Conventional growth *vs.* cold shock in wild-type and mutant *Neurospora*: A) Wild-type (Oak Ridge) *Neurospora* growth at 33°C, B) cold shock response in wild-type *Neurospora*, While many of the knockout strains tested displayed a morphological response to cold shock indistinguishable from that of wild-type, alternative morphologies were observed. These were classified into categories, examples of which are shown here. Examples of the alternate cold shock phenotypes displayed with the identity of the mutant shown as the example are shown: C) Burst: tips of growing hypha burst commonly (NCU02133, superoxide dismutase-1), D) Fail: a failure to display any morphological response to cold shock (NCU02636, peroxin-4), E) Thin: hyphal diameter narrows on cold shock (NCU03013, anchored cell wall protein-10), F) Dense: apical branching tighter than that normally displayed during cold shock (NCU07617, aconidiate-3), G) Cot-like: phenotype resembles that seen at the restrictive temperature of a temperature-sensitive colonial (cot) mutant strain. (NCU03901, peroxin-14), H) Weak: apical branching during cold shock which is less dense than normally observed (NCU01408, COP9 signalosome-3). Combinations of the above were sometimes observed as noted in Table 1.

Homeostasis in the face of temperature changes and more specifically the response to cold shock has been extensively studied in bacterial systems for over 20 years. The effect of cold shock is manifest in multiple cellular systems including: membrane rigidity (Shivaji & Prakash 2010), stability of secondary structures in DNA/RNA (Phadtare 2004), efficiency of protein folding (Phadtare 2004) and ribosome function (Gualerzi *et al.* 2011). While much remains to be described in these systems, cold shock appears to result in a multi-stage response (Phadtare 2004). First, a lag period in which growth and translation of proteins generally cease. This is followed by an adjustment phase in which specific cold-shock proteins which compensate for the changes brought on by the cold are preferentially translated (Giuliodori *et al.* 2004). In the final stage, growth continues otherwise normally, but at a reduced rate. DNA microarray transcription profiling of the cold shock response in *E. coli* by Phadtare and Inouye (2004) has shown that several hundred genes respond to cold shock, either being transiently induced/repressed or showing prolonged induction/repression. Analogous responses to cold shock and/or cold acclimation have been observed in diverse organisms including plants (Guy 1999) and animals (Canclini & Esteves 2007). Attempts to uncover cold shock proteins in fungi (Fang and St Leger 2010) have met with mixed success.

It is tempting to draw parallels between what is known about cold shock in bacterial systems and the observed response of *Neurospora* to similar cold shocks. Many of the systems affected during bacterial cold shock would be expected to impact fungal tip growth and branching (*e.g.*, membrane fluidity). In addition, the nature and timing of the two responses are similar. Both can be adjusted by changing the intensity of the cold shock with more mild shocks (lower temperature differences) producing more mild responses and more severe shocks (larger temperature differences) producing more severe responses. Furthermore, the dynamics of the responses parallel each other. In each, there is a multistage response. There is an initial response which is transient in nature, followed by a more long-term response which largely represents a return to normal growth.

The hypothesis of this project was that the observed cold shock response of *Neurospora* is a consequence of a cellular response homologous to that induced by cold shock in bacteria. Under this hypothesis, the observed, transient morphological changes are a consequence of the fungal cell adjusting itself to growth in the cold via a manner which is shared in common with simpler organisms. This hypothesis was tested by screening *Neurospora* knockout strains impacting genes homologous to those identified in *E. coli* which alter their expression patterns in response to cold shock. In addition, a broader collection of selected knockout strains were screened to identify additional genes which play a role in the cold shock response and thus cold acclimatization. Together, the results of this screen provides the first molecular underpinning to the cold shock response in *Neurospora*,

## Materials and Methods

### The *Neurospora* targeted deletion collection

As part of the *Neurospora* Genome Project, a collection of strains containing disruptions in presumptive genes was constructed (Colot *et al.* 2006). Strains representing deletions of most of the genes of the *Neurospora* genome are available from the Fungal Genetics Stock Center (McCluskey 2003) which supplied the knockout strains for this study. The FGSC supplied the knockout strains at a reduced fee in order to support undergraduate research. As each deletion strain has been altered in a single, previously identified, presumptive gene – going from phenotype to sequence is greatly simplified.

The accession numbers listed in Tables 1 and 2 represent the locus number of the gene subject to inactivation in the knockout strain under test. Every annotated gene in *Neurospora crassa* has been assigned a locus number of the form NCU#####. The gene identities reported in the tables are those associated with the genes as annotated on the FungiDB database as of July 2017: fungidb.org/fungidb/. The gene identities reported are based solely on the annotations currently associated with those strains and have not been independently confirmed by the authors of this study. Gene Ontologies reported are those determined by pantherdb.org (Mi *et al.* 2017) as of December 2017.

**Table 1:** Of 344 knockouts screened 118 were observed to alter the phenotype of the cold shock response. For each knockout strain tested (“ID”/NCU#####) we report the Cold Shock phenotype, the annotated gene function and gene abbreviation, the set of mutants the knockout came from (*E. coli* cold shock mutant ortholog, the Morphological or Hyphal growth plates from the FGSC, or the Transcription Factor plates from the FGSC) and the Gene Ontology categorizations for both Molecular Function and Biological Process. FGSC# is the strain number at the Fungal Genetics Stock Center

**ID**	**FGSC#**	**CS Phenotype**	**Gene Function**	**Gene**	**Knockout set**	**GO: Molecular Function**	**GO: Biological Process**
NCU03938	11228	burst	alternative oxidase-5	*aod-5*	Morph/Hyph		
NCU03070	11109	burst	hypothetical protein		Transc Factors	Binding	Biological Regulation
NCU01782	11375	burst	Ras guanyl-nucleotide exchange factor RasGEF		*E. coli* CS Orth		Cellular Process
NCU02133	11215	burst	superoxide dismutase-1	*sod-1*	Morph/Hyph	antioxidant/binding/catalytic Activity	Cellular Process/Response to Stimulus
NCU01213	11205	burst	superoxide dismutase-2	*sod-2*	*E. coli* CS Orth	Binding	Developmental Process
NCU03623	11226	burst	ubiquitin-conjugating enzyme E		Morph/Hyph	Binding	Cellular Process/Metabolic Process
NCU04242	11230	burst/dense	period-6	*prd-6*	Morph/Hyph	Binding/Catalytic Activity	Biological Regulation/Cellular Process/Metabolic Process
NCU07728	11268	burst/thin	siderophore regulation	*sre*	Morph/Hyph		
NCU03901	11305	cot-like	peroxin 14	*pex14*	Morph/Hyph	Binding	Cellular Component Organization or Biogenisis/ Cellular Process/Localization/Metabolic Process
NCU07617	11254	dense	aconidiate-3	*acon-3*	Morph/Hyph		Biological Regulation/Developmental Process/ Reproduction
NCU05410	16183	dense	arginine-5	*arg-5*	*E. coli* CS Orth	Binding	Cellular Process/Metabolic Process
NCU02114	11571	dense	G1/S-specific cyclin Cln1		Morph/Hyph	Binding	Cellular Process
NCU00144	11340	dense	hypothetical protein		Transc Factors		
NCU03120	11036	dense	hypothetical protein		Transc Factors		
NCU03356	11128	dense	hypothetical protein		Transc Factors		
NCU03417	11083	dense	hypothetical protein		Transc Factors		
NCU03905	11131	dense	hypothetical protein		Transc Factors		
NCU03962	11112	dense	hypothetical protein		Transc Factors	Binding	Cellular Process/Metabolic Process
NCU06990	11032	dense	hypothetical protein		Transc Factors		
NCU01154	11127	dense	submerged protoperithecia-1	*sub-1*	Transc Factors		
NCU04899	17402	dense	tricarboxylic acid-15	*tca-15*	*E. coli* CS Orth	Catalytic Activity	Metabolic Process
NCU03415	12921	fail	aldehyde dehydrogenase	*CBS-3*	*E. coli* CS Orth		
NCU11289	23565	fail	aldo-keto reductase		*E. coli* CS Orth	Catalytic Activity/transporter Activity	
NCU00097	11110	fail	BEAK-1	*bek-1*	Transc Factors		
NCU02017	11108	fail	CBF/NF-Y family transcription factor	*ada-2*	Transc Factors		
NCU00056	21444	fail	condensing enzyme with mitochondrial function	*cem-1*	*E. coli* CS Orth		
NCU00467	11284	fail	COP9 signalosome-5	*csn-5*	Morph/Hyph	Binding	Metabolic Process
NCU06068	11063	fail	fungal specific transcription factor	*col-25*	Transc Factors		
NCU07788	11031	fail	fungal specific transcription factor	*col-26*	Transc Factors		
NCU07945	11056	fail	fungal specific transcription factor	*tah-4*	Transc Factors		
NCU07947	13023	fail	glycolipid transfer protein HET-C2		Morph/Hyph		Localization/Metabolic Process
NCU05927	20010	fail	GTP-binding protein GUF1	*GTP-7*	*E. coli* CS Orth		
NCU00528	12080	fail	hyphal anastomosis-4	*ham-4*	*E. coli* CS Orth		
NCU07561	11114	fail	hypothetical protein		Transc Factors		
NCU09120	11964	fail	lysine-specific histone demethylase Aof2		*E. coli* CS Orth	Binding	Cellular Process/Metabolic Process
NCU09830	11263	fail	menadione-induced gene-12	*mig-12*	Morph/Hyph	Catalytic Activity	Cellular Process/Metabolic Process
NCU09842	11321	fail	mitogen activated protein kinase-1	*mak-1*	Morph/Hyph	Catalytic Activity/signal transducer activity	Biological Regulation/ Cellular Process/ Response to Stimulus/ Metabolic Process
NCU03314	11296	fail	mob2-like-a	*mob-2a*	Morph/Hyph	Binding/Catalytic Activity	Cellular Process
NCU09975	14572	fail	multidrug resistance protein 3		*E. coli* CS Orth		
NCU08294	11007	fail	nitrogen assimilation transcription factor nit-4	*nit-4*	Transc Factors		
NCU03277	11333	fail	peroxin 10	*pex10*	Morph/Hyph		Cellular Component Organization or Biogenisis/Cellular Process/Localization
NCU02636	11221	fail	peroxin 4	*pex4*	Morph/Hyph		
NCU01004	22657	fail	phosphatidylserine decarboxylase proenzyme	*CHOL-15*	*E. coli* CS Orth	Catalytic Activity	Cellular Process/Metabolic Process
NCU07832	20832	fail	pre-mRNA processing splicing factor 8	*msp-39*	*E. coli* CS Orth	Catalytic Activity	Cellular Component Organization or Biogenisis/Cellular Process/Metabolic Process
NCU06028	11034	fail	quinic acid utilization activator	*qa-1F*	Transc Factors		
NCU06205	11372	fail	regulator of conidiation-1	*rco-1*	Morph/Hyph		
NCU06145	12557	fail	RING-6	*RING-6*	Morph/Hyph		
NCU02214	11068	fail	TAH-2	*tah-2*	Transc Factors		
NCU10008	22177	fail	tricarboxylic acid-14	*tca-14*	*E. coli* CS Orth		Cellular Process/Metabolic Process
NCU02356	11712	fail	white collar 1	*wc-1*	Transc Factors	Binding	
NCU02173	11440	fail	zinc finger transcription factor-52	*znf-52*	Transc Factors		
NCU05591	11239	thin	ABC transporter CDR4		Morph/Hyph	Catalytic Activity/transporter Activity	Cellular Process/Metabolic Process
NCU03013	11223	thin	anchored cell wall protein-10	*acw-10*	Morph/Hyph	antioxidant/binding/catalytic Activity	Cellular Process/Response to Stimulus
NCU02333	11217	thin	arginase-1	*aga-1*	Morph/Hyph	Binding/Catalytic Activity	Cellular Process/Metabolic Process
NCU03184	11357	thin	C2H2 conidiation transcription factor FlbC		Morph/Hyph		
NCU07075	11249	thin	calcium exchanger	*cax*	Morph/Hyph	transporter activity	Biological Regulation/ Cellular Process
NCU05770	11532	thin	catalase-2	*cat-2*	*E. coli* CS Orth	antioxidant/binding	Cellular Process/Response to Stimulus/Metabolic Process
NCU05051	11097	thin	COL-23	*col-23*	Transc Factors		
NCU00830	11286	thin	ctr copper transporter	*tcu-1*	Morph/Hyph		
NCU08216	22525	thin	cystathionine beta-synthase	*MET-11*	*E. coli* CS Orth	Binding	Cellular Process/Metabolic Process
NCU03076	11294	thin	delta-1-pyrroline-5-carboxylate dehydrogenase		Morph/Hyph		
NCU08968	22160	thin	dimethyladenosine transferase		*E. coli* CS Orth		Cellular Component Organization or Biogenisis/ Cellular Process/Metabolic Process
NCU01772	22283	thin	DNA-directed RNA polymerase III polypeptide	*rpo-10*	*E. coli* CS Orth		Cellular Process/Metabolic Process
NCU02542	11220	thin	embden-meyerhof pathway-1	*emp-1*	Morph/Hyph	Catalytic Activity	Biological Regulation/ Cellular Process/ Metabolic Process
NCU01744	22231	thin	enhancer-2 of am	*en(am)-2*	*E. coli* CS Orth	Catalytic Activity	Cellular Process/Metabolic Process
NCU04264	11232	thin	extracellular developmental signal biosynthesis protein FluG		Morph/Hyph	Binding	Cellular Process/Metabolic Process
NCU04140	11562	thin	FK506 resistant-2	*fkr-2*	*E. coli* CS Orth	Binding/Catalytic Activity	Cellular Process/Metabolic Process
NCU09930	21617	thin	folic acid synthesis protein	*fol-9*	*E. coli* CS Orth	transporter activity	Cellular Process/Metabolic Process
NCU05606	13744	thin	glucosidase 2 subunit beta	*GHX-4*	*E. coli* CS Orth	Binding/Catalytic Activity	Biological Regulation/ Metabolic Process
NCU01528	22515	thin	glyceraldehyde-3-phosphate dehydrogenase-1	*gpd-1*	*E. coli* CS Orth	Catalytic Activity	Metabolic Process
NCU06005	13543	thin	glycerol kinase	*GLK-1*	*E. coli* CS Orth		
NCU02630	11952	thin	heat shock protein 78	*hsp78*	*E. coli* CS Orth		
NCU07156	20700	thin	histidine-6	*his-6*	*E. coli* CS Orth		
NCU02556	11840	thin	histone acetyl transferase-2	*hat-2*	*E. coli* CS Orth		Cellular Component Organization or Biogenisis/ Cellular Process/ Metabolic Process
NCU01629	11102	thin	hypothetical protein		Transc Factors		
NCU04669	11307	thin	hypothetical protein homologous to Bactericidal permeability-increasing protein		Morph/Hyph		
NCU04561	11136	thin	melanization defective-1	*mld-1*	Transc Factors		
NCU09767	18564	thin	membrane transporter		*E. coli* CS Orth		
NCU04791	18772	thin	menadione-induced gene-10	*mig-10*	*E. coli* CS Orth		
NCU05151	13482	thin	phosphoketolase	*PHK-1*	*E. coli* CS Orth		
NCU06342	20075	thin	phospholipase D	*PLA-5*	*E. coli* CS Orth		Cellular Process/Localization/Locomotion
NCU05295	11593	thin	proteasome catalytic alpha-5	*pca-5*	Morph/Hyph		Cellular Process/Metabolic Process
NCU09366	11603	thin	proteasome catalytic beta-6	*pcb-6*	Morph/Hyph	Catalytic Activity	Cellular Process/Metabolic Process
NCU01613	11291	thin	protoperithecia-2	*pp-2*	Morph/Hyph	Catalytic Activity	Biological Regulation/Cellular Component Organization or Biogenisis/ Cellular Process/ Metabolic Process
NCU02260	11586	thin	regulatory particle, ATPase-like-3	*rpt-3*	Morph/Hyph		
NCU02055	13283	thin	uridine nucleosidase Urh1	*NUS-1*	*E. coli* CS Orth		
NCU07705	11029	thin/fail	C6 finger domain-containing protein	*clr-1*	Transc Factors		
NCU08000	11005	thin/fail	cutinase transcription factor 1 alpha	*far1*	Transc Factors		
NCU05536	11027	thin/fail	hypothetical protein		Transc Factors	Binding/Catalytic Activity	Cellular Component Organization or Biogenisis/ Cellular Process/Response to Stimulus/ Metabolic Process
NCU08651	11012	thin/fail	zinc binuclear cluster-type protein	*col-27*	Transc Factors		
NCU07732	14014	thin/fail	arginine-2	*arg-2*	*E. coli* CS Orth	Binding/Catalytic Activity	Cellular Process/Metabolic Process
NCU04117	21178	thin/fail	ATP-dependent permease MDL2	*ABC-7*	*E. coli* CS Orth		
NCU06659	12287	thin/fail	GTP-binding protein	*GTP-3*	*E. coli* CS Orth		Cellular Process/Metabolic Process
NCU08693	14197	thin/fail	heat shock protein 70-5	*hsp70-5*	*E. coli* CS Orth	Binding	Cellular Process/Response to Stimulus/Metabolic Process
NCU10760	12539	thin/fail	jumonji domain-containing protein 5		*E. coli* CS Orth		
NCU08858	14492	thin/fail	MFS alpha-glucoside transporter	*SUT-1*	*E. coli* CS Orth	transporter activity	Cellular Process/ Localization
NCU00793	15944	thin/fail	trehalose phosphate synthase	*GT20-2*	*E. coli* CS Orth	Catalytic Activity	Cellular Process/Response to Stimulus/Metabolic Process
NCU08336	22591	thin/fail	tricarboxylic acid-12	*tca-12*	*E. coli* CS Orth	Catalytic Activity	Cellular Process/ Localization/Metabolic Process
NCU00771	19376	thin/fail	UBX domain-containing protein 7		*E. coli* CS Orth		
NCU04583	12407	weak	acetyltransferase		*E. coli* CS Orth	Catalytic Activity	
NCU00499	11120	weak	all development altered-1	*ada-1*	Transc Factors		
NCU00567	22271	weak	arginine-6	*arg-6*	*E. coli* CS Orth	Binding	Cellular Process/Metabolic Process
NCU04303	16296	weak	asparagine synthetase 2	*asn-1*	*E. coli* CS Orth	Binding/Catalytic Activity	Cellular Process/Metabolic Process
NCU00919	16502	weak	ATP-dependent RNA helicase rok-1	*drh-16*	*E. coli* CS Orth		
NCU08933	23868	weak	cellular nucleic acid-binding protein		*E. coli* CS Orth		
NCU01408	11275	weak	COP9 signalosome-3	*csn-3*	Morph/Hyph		Cellular Process/Metabolic Process
NCU01625	15732	weak	DNA repair helicase RAD3	*DNR-10*	*E. coli* CS Orth	Binding/Catalytic Activity	Cellular Process/Metabolic Process
NCU07027	20154	weak	glycogen phosphorylase	*GYP-1*	*E. coli* CS Orth	Binding/Catalytic Activity	Cellular Process/Metabolic Process
NCU06523	23841	weak	glycosylhydrolase family 13-4	*gh13-4*	*E. coli* CS Orth	Catalytic Activity	Metabolic Process
NCU01589	13671	weak	heat shock protein 60	*hsp60*	*E. coli* CS Orth	Binding	Cellular Component Organization or Biogenisis/ Cellular Process/ Metabolic Process
NCU05909	11104	weak	hypothetical protein		Transc Factors		
NCU08439	15564	weak	leptomycin B resistance protein pmd1	*ABC-2*	*E. coli* CS Orth	transporter activity	Cellular Process/Metabolic Process
NCU00565	18702	weak	lipoic acid synthetase	*LIA-1*	*E. coli* CS Orth	Catalytic Activity	Cellular Process/Metabolic Process
NCU04339	16454	weak	ribokinase	*RIK-8*	*E. coli* CS Orth	Catalytic Activity	Cellular Process/Metabolic Process
NCU03894	11325	weak	serine/threonine protein kinase-4	*stk-4*	Morph/Hyph	Binding/signal transducer activity	Biological Regulation/Cellular Process/Developmental Process/ Response to Stimulus
NCU06017	13547	weak	thiosulfate sulfurtransferase	*TST-1*	*E. coli* CS Orth		Localization/Response to Stimulus/Metabolic Process
NCU10053	21996	weak	thymidylate synthase	*pyr-8*	*E. coli* CS Orth		
NCU08658	11059	weak	zinc finger transcription factor-50	*znf-50*	Transc Factors		

**Table 2:** Of knockouts screened, 226 presented no change to the cold shock morphology. Columns are the same as for Table 1

**ID**		**Gene Function**	**Gene**	**Knockout set**	**GO: Molecular Function**	**GO: Biological Process**
NCU00017	11075	hypothetical protein		Transc Factors		
NCU00019	11437	Fork head protein homolog 1	*FKH1*	Transc Factors		
NCU00038	11483	zinc finger transcription factor-32	*znf-32*	Transc Factors		
NCU00081	15983	DNA topoisomerase 3-beta	*dnt-3*	*E. coli* CS Orth		
NCU00090	11397	pH-response transcription factor pacC/RIM101	*pacc-1*	Transc Factors		
NCU00105	15796	ribosome biogenesis-58	*rbg-58*	Morph/Hyph		Cellular Component Organization or Biogenisis
NCU00135	16021	Phosphatidyl synthase, phosphatidyl synthase, variant 1	*gpl-1*	Morph/Hyph		Cellular Process/Metabolic Process
NCU00157	11282	COP9 signalosome-1	*csn-1*	Morph/Hyph		
NCU00204	12199	hypothetical protein		Morph/Hyph		
NCU00217	11020	hypothetical protein		Transc Factors		
NCU00233	11117	glycosyl hydrolase family 16-15	*gh16-15*	Transc Factors		
NCU00285	11118	hypothetical protein		Transc Factors		
NCU00289	11085	tall aerial hyphae-1	*tah-1*	Transc Factors		
NCU00329	11119	vegetative asexual development-1	*vad-1*	Transc Factors		
NCU00355	11202	catalase-3	*cat-3*	Morph/Hyph	Antioxidant Activity/Binding/Catalytic Activity	Response to Stimulus/Cellular Process/Metabolic Process
NCU00396	11612	pre-mRNA-splicing factor rse-1	*msp-5*	Morph/Hyph	Binding	Cellular Process/Metabolic Process
NCU00406	11323	velvet	*vel*	Morph/Hyph	Binding/Signal Transducer Activity/Catalytic Activity	Biological Regulation/Developmental Process/ Response to Stimulus/Cellular Process
NCU00554	16113	Aspartate-semialdehyde dehydrogenase	*hom-1*	Morph/Hyph		
NCU00609	16119	initiation-specific alpha-1,6-mannosyltransferase	*och-1*	Morph/Hyph	Catalytic Activity	Cellular Process/Metabolic Process
NCU00631	11738	chromatin remodelling factor 9-1	*crf9-1*	Transc Factors		
NCU00634	16123	Ribosomal protein L14	*crp-47*	Morph/Hyph	Structural Molecule Activity	Cellular Component Organization or Biogenisis
NCU00694	11103	hypothetical protein		Transc Factors		
NCU00749	11438	conidiation at high carbon dioxide-1	*chc-1*	Transc Factors		
NCU00768	15724	mRNA binding post-transcriptional regulator		Morph/Hyph		
NCU00808	11122	zinc finger transcription factor-48	*znf-48*	Transc Factors		
NCU00810	11285	Beta-galactosidase	*gh2-3*	Morph/Hyph	Catalytic Activity	Cellular Process/Metabolic Process
NCU00824	11614	histone deacetylase-3	*hda-3*	Morph/Hyph	Binding/Catalytic Activity	Biological Regulation/Cellular Compoonent Organization or Biogenisis/ Cellular Process
NCU00902	11124	zinc finger white collar protein WC2	*wc-2*	Transc Factors		
NCU00923	11273	topogenesis of outer membrane beta barrel protein 37	*tob37*	Morph/Hyph		
NCU00945	11064	fungal specific transcription factor	*col-20*	Transc Factors		
NCU00959	16505	succinate dehydrogenase iron-sulfur protein	*tca-10*	*E. coli* CS Orth		Cellular Process/Metabolic Process
NCU01020	13009	hypothetical protein		Morph/Hyph		
NCU01033	11204	hypothetical protein related to regulatory protein wetA		Morph/Hyph	Binding	
NCU01037	13038	hypothetical protein		Morph/Hyph		
NCU01097	11038	hypothetical protein		Transc Factors		
NCU01122	11125	hypothetical protein		Transc Factors		
NCU01181	11287	acyl-CoA dehydrogenase family member 11	*acd-3*	Morph/Hyph		
NCU01197	11288	cell wall biogenesis protein phosphatase Ssd1	*gul-1*	Morph/Hyph		
NCU01213	11206	superoxide dismutase-2	*sod-2*	Morph/Hyph	Antioxidant Activity/Binding/Catalytic Activity	Developmental Process
NCU01225	11207	ubiquitin conjugating enzyme - 13	*uce-13*	Morph/Hyph		
NCU01312	11209	myb-like DNA-binding protein myb-1	*rca-1*	Morph/Hyph		
NCU01368	11582	proteasome component ^11^C	*pcb-4*	Morph/Hyph	Catalytic Activity	Cellular Process/Metabolic Process
NCU01478	11002	fungal specific transcription factor domain-containing protein		Transc Factors		
NCU01642	11211	hypothetical protein homologous to Neurofibromin		Morph/Hyph		
NCU01833	11213	Two-component histidine kinase CHK-1	*nik-2*	Morph/Hyph		
NCU01994	11342	transcription factor-1	*tcf-1*	Transc Factors		
NCU02057	11214	autoinducer 2 sensor kinase/phosphatase luxQ		Morph/Hyph		
NCU02094	11060	vegetative asexual development-2	*vad-2*	Transc Factors	Binding	Cellular Process/Metabolic Process
NCU02111	11611	myosin-5	*myo-5*	Morph/Hyph	Binding/Structural Molecule Activity/Catalytic Activity	Cellular Component Organization or Biogenisis/Localization Process/ Cellular Process
NCU02142	11071	hypothetical protein		Transc Factors		
NCU02160	11525	small GTPase RAC	*rac-1*	Morph/Hyph	Binding/Signal Transducer Activity/Catalytic Activity	Biological Regulation/Cellular Compoonent Organization or Biogenisis/ Developmental Process/ Response to Stimulus/ Cellular Process/Metabolic Process
NCU02226	16056	methylthioribose-1-phosphate isomerase	*met-23*	Morph/Hyph	Catalytic Activity	Cellular Process/Metabolic Process
NCU02250	16168	ATP synthase subunit ATP9	*oli*	Morph/Hyph	Transporter Activity/Catalytic Activity	Cellular Process/Metabolic Process
NCU02265	11554	period clock protein FRQ	*frq*	Morph/Hyph		
NCU02307	11054	hypothetical protein		Transc Factors		
NCU02387	11219	nuclear import and export protein Msn5		Morph/Hyph	Binding/Transporter Activity	Biological Regulation/Localization Process/Cellular Process
NCU02406	16076	nuclear protein		Morph/Hyph	Binding	Cellular Component Organization or Biogenisis
NCU02498	11289	Cullin-3	*cul-3*	Morph/Hyph	Binding	Cellular Process/Metabolic Process
NCU02576	11072	zinc finger transcription factor-39	*znf-39*	Transc Factors		
NCU02604	11659	U3 small nucleolar RNA-associated protein 10	*rbg-7*	Morph/Hyph	Binding	Biological Regulation/Cellular Compoonent Organization or Biogenisis/Cellular Process/Metabolic Process
NCU02639	16474	Argininosuccinate synthase	*arg-1*	*E. coli* CS Orth	Catalytic Activity	Cellular Process/Metabolic Process
NCU02666	11344	zinc finger transcription factor-58	*znf-58*	Transc Factors		
NCU02671	11345	cutinase G-box binding protein	*msn-1*	Transc Factors		
NCU02699	11347	zinc finger transcription factor-14	*znf-14*	Transc Factors		
NCU02712	15714	acetate-10	*ace-10*	*E. coli* CS Orth		
NCU02713	11348	conidial separation-1	*csp-1*	Transc Factors	Binding	Cellular Process/Metabolic Process
NCU02724	11349	transcription factor-21	*tcf-21*	Transc Factors		
NCU02752	11015	zinc finger transcription factor-47	*znf-47*	Transc Factors		
NCU02768	11090	transcription factor-20	*tcf-20*	Transc Factors		
NCU02794	11293	Fso1	*so*	Morph/Hyph		
NCU02826	11529	sodium/calcium exchanger protein	*trm-16*	Morph/Hyph	Transporter Activity	
NCU02896	11070	all development altered-3	*ada-3*	Transc Factors		
NCU02934	11003	hypothetical protein		Transc Factors		
NCU02948	16325	non-anchored cell wall protein-4	*ncw-4*	*E. coli* CS Orth	Catalytic Activity	
NCU02957	11350	hypothetical protein		Transc Factors		
NCU02994	11353	hypothetical protein		Transc Factors		
NCU03033	11725	transcription factor-26	*tcf-26*	Transc Factors	Binding	Biological Regulation/Response to Stimulus
NCU03043	11224	C2H2 finger domain-containing protein FlbC	*acon-4*	Transc Factors		
NCU03073	11107	DNA polymerase epsilon, subunit D	*pole-4*	Transc Factors		
NCU03077	11356	hypothetical protein		Transc Factors		
NCU03096	12860	bromodomain associated domain-containing protein		Morph/Hyph		
NCU03110	11024	hypothetical protein		Transc Factors		
NCU03125	11279	NIMA-interacting protein TinC		Morph/Hyph		
NCU03164	11225	two-component system response regulator		Morph/Hyph		
NCU03206	11486	zinc finger transcription factor-22	*znf-22*	Transc Factors		
NCU03244	11360	WD repeat protein		Transc Factors		
NCU03281	11276	transport of copper-2	*tcu-2*	Morph/Hyph		
NCU03320	11058	all development altered-4	*ada-4*	Transc Factors		
NCU03479	12931	endoribonuclease ysh-1	*paa-5*	Morph/Hyph	Binding/Catalytic Activity	Metabolic Process
NCU03489	11095	colonial-21	*col-21*	Transc Factors		
NCU03576	13043	conidiophore development protein hymA	*hym-1*	Morph/Hyph	Binding	
NCU03593	11129	homeobox domain-containing protein	*kal-1*	Transc Factors		
NCU03643	11049	fatty acid regulation-2	*far-2*	Transc Factors		
NCU03669	11658	AdoMet-dependent rRNA methyltransferase spb1	*rmt-3*	*E. coli* CS Orth	Catalytic Activity	Cellular Component Organization or Biogenisis/ Cellular Process/Metabolic Process
NCU03686	11076	oxidase assembly protein 2	*tah-3*	Transc Factors		
NCU03699	11130	zinc finger transcription factor-13	*znf-13*	Transc Factors		
NCU03702	11836	rRNA 2’-O-methyltransferase fibrillarin	*rbg-16*	Morph/Hyph		
NCU03725	11309	vegetative incompatibility blocked-1	*vib-1*	Morph/Hyph	Binding	Biological Regulation/Cellular Process/Metabolic Process
NCU03931	11053	all development altered-5	*ada-5*	Transc Factors		
NCU04001	11073	female fertility-7	*ff-7*	Transc Factors	Catalytic Activity	
NCU04096	11317	serine/threonine-protein kinase 3	*prk-9*	Morph/Hyph	Binding/Signal Transducer Activity/Catalytic Activity	Biological Regulation/Developmental Process/ Multicellular Organismal Process/Response to Stimulus/Cellular Process
NCU04142	11468	heat shock protein 80	*hsp80*	*E. coli* CS Orth		Response to Stimulus/ Metabolic Process
NCU04179	11132	C2H2 transcription factor	*sah-1*	Transc Factors		
NCU04211	11133	hypothetical protein		Transc Factors		
NCU04302	11233	ubiquitin-conjugating enzyme E	*nup-22*	Morph/Hyph		
NCU04359	11045	hypothetical protein		Transc Factors		
NCU04390	11134	fungal specific transcription factor	*col-22*	Transc Factors		
NCU04513	11234	ubiquitin conjugating enzyme Ubc14	*uce-14*	Morph/Hyph	Catalytic Activity	Metabolic Process
NCU04533	11298	DUF1881 domain-containing protein	*app*	Morph/Hyph		
NCU04619	11137	hypothetical protein		Transc Factors		
NCU04628	11138	hypothetical protein		Transc Factors		
NCU04731	11139	Sterol regulatory element binding protein sah-2	*sah-2*	Transc Factors		
NCU04733	11737	UvrD/REP helicase	*mus-50*	*E. coli* CS Orth		
NCU04834	11236	sensor histidine kinase/response regulator	*phy-1*	Morph/Hyph		
NCU04851	11089	hypothetical protein		Transc Factors		
NCU04866	11022	all development altered-6	*ada-6*	Transc Factors		
NCU05046	11237	calcium-transporting ATPase 3	*ena-1*	Morph/Hyph	Transporter Activity/Catalytic Activity	Cellular Process/Metabolic Process
NCU05210	11444	postreplication repair E3 ubiquitin-protein ligase rad-18	*uvs-2*	Transc Factors	Catalytic Activity	Response to Stimulus/Cellular Process/Metabolic Process
NCU05242	11364	zinc finger transcription factor-25	*znf-25*	Transc Factors		
NCU05250	11492	nuclear division-76	*div-76*	Transc Factors	Binding	Biological Regulation/Cellular Compoonent Organization or Biogenisis/Localization Process/Response to Stimulus/Cellular Process/Metabolic Process
NCU05294	11074	zinc finger transcription factor-40	*znf-40*	Transc Factors		
NCU05383	11019	fungal specific transcription factor	*col-24*	Transc Factors		
NCU05411	11040	pathway-specific nitrogen regulator		Transc Factors		
NCU05637	11365	hypothetical protein		Transc Factors		
NCU05767	11051	zinc finger transcription factor-10	*znf-10*	Transc Factors		
NCU05790	11241	phytochrome-like histidine kinase 2	*phy-2*	Morph/Hyph		
NCU05854	11314	hypothetical protein		Morph/Hyph		
NCU05858	11242	fatty acid oxygenase	*fam-2*	Morph/Hyph	Catalytic Activity	
NCU05891	11904	arid/bright domain-containing protein		Morph/Hyph	Binding	Biological Regulation/Cellular Process/Metabolic Process
NCU05956	11310	Beta-galactosidase	*gh2-2*	Morph/Hyph	Catalytic Activity	Cellular Process/Metabolic Process
NCU05993	11078	hypothetical protein		Transc Factors		
NCU05994	11025	transcription factor-10	*tcf-10*	Transc Factors	Binding	Cellular Component Organization or Biogenisis/Localization Process/Response to Stimulus/Cellular Process/Metabolic Process
NCU06049	12674	DNA damage response protein RcaA	*nbs1*	Morph/Hyph	Binding/Catalytic Activity	Biological Regulation/Cellular Compoonent Organization or Biogenisis/Response to Stimulus/ Cellular Process/Metabolic Process
NCU06145	12558	RING-6		Morph/Hyph		
NCU06173	11366	hypothetical protein		Transc Factors		
NCU06175	11244	Peroxisomal membrane protein	*pex3*	Morph/Hyph		
NCU06186	11369	hypothetical protein		Transc Factors		
NCU06205	11371	transcriptional repressor rco-1	*rco-1*	Transc Factors		
NCU06213	11373	zinc finger transcription factor-9	*znf-9*	Transc Factors		
NCU06265	11245	Hyphal anastamosis-13 protein	*ham-13*	Morph/Hyph		
NCU06407	11017	zinc finger transcription factor 1	*vad-3*	Transc Factors		
NCU06411	11116	vegetative asexual development-4	*vad-4*	Transc Factors	Binding/Catalytic Activity	Metabolic Process
NCU06419	11319	map kinase kinase	*mek-1*	Morph/Hyph	Binding/Signal Transducer Activity/Catalytic Activity	Biological Regulation/Developmental Process/ Response to Stimulus/Cellular Process
NCU06429	11835	alpha-actinin		Morph/Hyph		
NCU06440	11595	proteasome component PRE6	*pca-4*	Morph/Hyph	Catalytic Activity	Cellular Process/ Metabolic Process
NCU06454	15833	Rho-type GTPase	*cdc42*	Morph/Hyph	Binding/Signal Transducer Activity/Catalytic Activity	Biological Regulation/Cellular Compoonent Organization or Biogenisis/ Developmental Process/ Response to Stimulus/ Cellular Process/Metabolic Process
NCU06503	11377	zinc finger transcription factor-24	*znf-24*	Transc Factors		
NCU06531	11312	hypothetical protein		Morph/Hyph		
NCU06605	11184	DNA damage-binding protein 1	*dim-8*	Morph/Hyph	Binding	Response to Stimulus/Cellular Process/Metabolic Process
NCU06650	11247	secretory phospholipase A2	*spp-3*	Morph/Hyph		
NCU06656	11013	transcriptional activator protein acu-15	*acu-15*	Transc Factors		
NCU06695	15946	cytochrome c oxidase polypeptide VI	*cox-6*	Morph/Hyph	Transporter Activity/Catalytic Activity	Cellular Process/Metabolic Process
NCU06714	12653	para-aminobenzoic acid synthetase	*pab-1*	Morph/Hyph		
NCU06744	11379	hypothetical protein		Transc Factors		
NCU06764	11597	20S proteasome subunit Y7	*pca-2*	Morph/Hyph	Catalytic Activity	Cellular Process/Metabolic Process
NCU06799	11001	fungal specific transcription factor	*vad-5*	Transc Factors		
NCU06845	12617	short chain dehydrogenase/reductase		Morph/Hyph		
NCU06910	15950	Cell wall integrity and stress response component 1	*wsc-1*	Morph/Hyph		
NCU06919	11105	hypothetical protein		Transc Factors		
NCU06971	11066	transcriptional activator xlnR	*xlr-1*	Transc Factors		
NCU07007	11006	submerged protoperithecia-2	*sub-2*	Transc Factors		
NCU07039	11381	GATA type zinc finger protein Asd4	*asd-4*	Transc Factors		
NCU07139	11055	BEAK-2	*bek-2*	Transc Factors		
NCU07221	11251	two-component system protein A	*hcp-1*	Morph/Hyph		
NCU07237	23704	hypothetical protein		*E. coli* CS Orth		
NCU07281	14469	glucose-6-phosphate isomerase	*gpi-1*	*E. coli* CS Orth	Catalytic Activity	Metabolic Process
NCU07374	11016	hypothetical protein		Transc Factors		
NCU07378	11252	serine threonine protein kinase	*stk-12*	Morph/Hyph	Catalytic Activity	Biological Regulation/Cellular Compoonent Organization or Biogenisis/Response to Stimulus/ Cellular Process/ Metabolic Process
NCU07379	11383	transcription factor-5	*tcf-5*	Transc Factors		
NCU07392	11041	transcriptional regulatory protein pro-1	*adv-1*	Transc Factors		
NCU07420	11844	eIF4A	*eif4A*	Morph/Hyph		
NCU07535	11094	SAH-3	*sah-3*	Transc Factors	Catalytic Activity	Cellular Process/ Metabolic Process
NCU07589	12409	acetyltransferase		Morph/Hyph		
NCU07591	12816	Integral membrane protein		Morph/Hyph		
NCU07605	11253	hypothetical protein		Morph/Hyph		
NCU07621	11301	zinc-regulated transporter 1	*tzn-1*	Morph/Hyph	Transporter Activity	Cellular Process
NCU07900	11446	hypothetical protein		Transc Factors		
NCU07952	11494	zinc finger transcription factor-37	*znf-37*	Transc Factors		
NCU08049	11047	hypothetical protein		Transc Factors		
NCU08050	15817	hypothetical protein		Morph/Hyph		
NCU08055	11269	zip-like-1	*zip-1*	Transc Factors	Binding	Response to Stimulus/Cellular Process/Metabolic Process
NCU08063	11092	kinetochore protein-18	*kpr-18*	Transc Factors	Catalytic Activity	Cellular Process/Metabolic Process
NCU08093	15976	hypothetical protein		Morph/Hyph	Transporter Activity/Catalytic Activity	Cellular Component Organization or Biogenisis/ Cellular Process/Metabolic Process
NCU08147	11256	Na or K P-type ATPase	*ph7*	Morph/Hyph	Transporter Activity/Catalytic Activity	Cellular Process/Metabolic Process
NCU08148	22001	H+/nucleoside cotransporter		*E. coli* CS Orth	Transporter Activity	Localization Process/Cellular Process
NCU08225	11303	high affinity nickel transporter nic1	*trm-34*	Morph/Hyph		
NCU08289	11100	DNA methylation modulator-2	*dmm-2*	Transc Factors	Binding	
NCU08290	20277	Ku70/Ku80 family protein	*mus-51*	*E. coli* CS Orth	Binding	Biological Regulation/Cellular Compoonent Organization or Biogenisis/Response to Stimulus/ Cellular Process/Metabolic Process
NCU08443	11061	hypothetical protein		Transc Factors		
NCU08516	20323	aldose 1-epimerase	*aep-1*	*E. coli* CS Orth	Catalytic Activity	Metabolic Process
NCU08634	11384	hypothetical protein		Transc Factors		
NCU08652	11009	hypothetical protein		Transc Factors		
NCU08726	11044	fluffy	*fl*	Transc Factors		
NCU08741	11300	Hyphal anastamosis protein 3	*ham-3*	Morph/Hyph		
NCU08744	11386	hypothetical protein		Transc Factors		
NCU08791	11258	catalase-1	*cat-1*	Morph/Hyph	Antioxidant Activity/Binding/Catalytic Activity	Response to Stimulus/Cellular Process/Metabolic Process
NCU08848	11043	hypothetical protein		Transc Factors		
NCU08875	11259	Cullin binding protein CanA		Morph/Hyph		Cellular Component Organization or Biogenisis/ Metabolic Process
NCU08891	11762	hypothetical protein		Transc Factors		Cellular Process
NCU08899	11048	hypothetical protein		Transc Factors		
NCU08901	11087	hypothetical protein		Transc Factors		
NCU08927	15707	dihydroceramide delta(4)-desaturase	*dcd*	Morph/Hyph	Catalytic Activity	Cellular Process/ Metabolic Process
NCU08992	15958	hypothetical protein		Morph/Hyph	Binding	Cellular Component Organization or Biogenisis/ Cellular Process/Metabolic Process
NCU09033	11390	zinc finger transcription factor-46		Transc Factors		
NCU09068	11392	nitrogen catabolic enzyme regulatory protein	*nit-2*	Transc Factors		
NCU09071	12000	AGC/NDR protein kinase	*dbf2*	Morph/Hyph	Catalytic Activity	Biological Regulation/Cellular Compoonent Organization or Biogenisis/Response to Stimulus/ Cellular Process/Metabolic Process
NCU09123	12547	Ca/CaM-dependent kinase-1	*camk-1*	Morph/Hyph		
NCU09201	11315	hypothetical protein		Morph/Hyph		
NCU09205	11096	nitrate assimilation regulatory protein nirA	*vad-6*	Transc Factors		
NCU09248	11496	transcription factor-27	*tcf-27*	Transc Factors		
NCU09252	11393	hypothetical protein		Transc Factors		
NCU09315	11448	phosphorus acquisition-controlling protein	*nuc-1*	Transc Factors		
NCU09333	11395	Zinc finger transcription factor ace-1	*ace-1*	Transc Factors		
NCU09364	11267	Hsp30-like protein	*hsp30*	Morph/Hyph		Response to Stimulus/ Metabolic Process
NCU09423	11261	secreted protein related to phopholipase A2		Morph/Hyph		
NCU09450	11604	26S proteasome regulatory subunit Rpn2	*rpn-2*	Morph/Hyph	Catalytic Activity	Cellular Process/Metabolic Process
NCU09494	11280	hypothetical protein		Morph/Hyph		
NCU09529	11098	hypothetical protein		Transc Factors	Binding	Cellular Process/Metabolic Process
NCU09549	11084	zinc finger transcription factor-51	*znf-51*	Transc Factors		
NCU09655	11272	hypothetical protein		Morph/Hyph		
NCU09739	11062	all development altered-7	*fld*	Transc Factors		
NCU09804	11080	zinc finger transcription factor-43	*znf-43*	Transc Factors		
NCU09829	11065	hypothetical protein		Transc Factors		
NCU09866	11264	thyroid hormone receptor interactor 12		Morph/Hyph		
NCU09882	11266	metacaspase-1A	*mcp-1*	Morph/Hyph		
NCU10006	11396	hypothetical protein		Transc Factors		

### Knockout sets selected to be subjected to screen

A screen of the entire library was determined to be impractical. We instead screened an abbreviated subsection of the library chosen to be more likely to yield positive responses. These fall into three basic sets.

The first set are knockouts of genes homologous to those which show altered transcription in *E. coli* when subjected to cold shock (Phadtare and Inouye 2004). The protein sequences of *E. coli* genes identified by were retrieved from the *E. coli* database (ecocyc.org/). These amino acid sequences were then fed into a BLAST search on the NIH NCBI site (blast.ncbi.nlm.nih.gov/Blast.cgi) with the output limited to *Neurospora* sequences in order to identify their nearest *Neurospora* homologs. These homologs were then searched on FungiDB to determine which had knockout strains available. From this final list, 68 were selected for screening in this study. This set was selected to determine the degree of relationship between the cold shock response in *E. coli* and *Neurospora*.

Second, two previously organized sets of knockouts generally associated with hyphal growth and morphology and available from the FGSC were included in this screen. One set (identified as “plate 29 – morphologicals” by the FGSC) contained strains with knockouts known to cause morphological changes. The second set (identified as “Hyphal Growth Set” by the FGSC) contained strains with knockouts in genes homologous to genes in yeast known to affect polar growth. A total of 131 strains from these two sets were screened.

The last set consists of knockouts of known transcription factors in *Neurospora*. This collection is available as a set from the Fungal Genetics Stock Center (McCluskey 2003). It was selected for this screen to determine which transcription factors play a role in signaling to the cell that cold adaptation genes must be activated. A total of 145 strains from this set were screened.

### Media

Media and culturing procedures were those described in Davis & deSerres (1970). Growth described as being on “minimal” was in plates containing Vogel’s minimal medium (Davis & deSerres 1970) with 2% agar.

### Screen

The selected knockout strains were subjected to a screen looking for altered responses to cold shock. Wild-type *Neurospora* progresses through a three-stage response following a shift into the cold. To induce the cold shock response, we initially grew strains at 33° and shifted to 4°. We selected 33° as our “normal” temperature as the cold shock response has previously been demonstrated to be dependent on the degree of the temperature shift the hypha are subjected to (Watters *et al.* 2000). The larger temperature shift used here would be expected to result in tighter branching during the apical phase. We decided this was desirable as it would make any variations from the normal cold shock response more visible and easier to identify in the screen. Strains were inoculated by dropping a suspension of conidia onto Vogel’s Minimal Medium and incubated overnight at 33°. The next morning plates were moved to 4°. After an overnight incubation at 4°, the strain’s response to cold shock was photographed and evaluated. Variations in the cold shock response from that of wild-type *Neurospora* were judged qualitatively. Knockouts were subjected to cold shock and photographed a minimum of three independent runs on separate days to assure consistency of the response within a strain.

### Photomicroscopy

Growing cultures were examined and photographed using a Motic 10MP digital camera attached to a Wolfe Beta Elite trinocular microscope. Photographs were taken of well separated, leading hyphae. All photomicrographs were taken using 40x magnification.

### Phenotypes scored

The morphology of strains following cold shock was scored visually by comparing collections of photographs of cold shock in a given strain to the response seen with a wild type strain (*Neurospora crassa* Oak Ridge). Those with altered responses were then further categorized visually into the groups reported in Table 1 “CS phenotype.”

### Undergraduate Student Involvement in Research

Valparaiso University is an undergraduate institution. All of the experiments reported here were conducted by undergraduate students under the supervision of the corresponding author. Students came to the lab under a variety of circumstances. Six of the student co-authors engaged the project as students in our Bio 496 (Independent Research) course in which students conduct research in the lab of a faculty member under their supervision. Two were upperclassmen working in the lab as paid assistants while being supported by a grant by the Indiana Space Grant Consortium (INSGC). The INSGC also supported a student from the local community college who contributed to this study. An additional student was supported by a separate grant from the INSGC with the purpose of bringing freshmen into research labs for a true research experience.

This project was chosen specifically to be one which would work well in the undergraduate university environment. The choice of organism as well as the project are suitable to a setting where funds are limited (or at times, unavailable). The study of morphology is one which students can easily grasp, and which they find relatively easy to score. Applying these questions to the knockout library allows us to take advantage of this tool and turn a quick screen into a collection of mostly identified gene functions associated with the trait. The work is technically straightforward, so undergraduate students can involve themselves with the actual conduct of the project after fairly little training in the basics of media preparation, sterile technique, basic microbiological techniques and the use of the microscope and camera.

The corresponding author was responsible for the design of the project. Undergraduates were then organized into teams incorporating both newer and older student researchers so the more experienced students could help guide the newer ones. Within these groups, students were responsible for dividing up aspects of the day-to-day activity of the project into segments and assigning individuals to be responsible for carrying out that day’s activity. This allowed them to dovetail the research activities into their normal class and work schedules. For example in a given week, one student would be responsible for making media, another for inoculating plates, and another for photomicroscopy. Scoring and categorization of the mutant phenotypes was conducted by students by examining photographs and confirmed by the corresponding author.

### Data availability

The authors state that all data necessary for confirming the conclusions presented in the article are represented fully within the article.

## Results and Discussion

During the initial study of the cold shock response in *Neurospora* (Watters *et al.* 2000), it was observed that two classical morphological mutants (most notably “granular” and “delicate”) produced altered responses to cold shock (not reported), demonstrating that mutants could be obtained which influenced this process. We chose to screen mutants from the *Neurospora* knockout library for their cold shock response in order to provide a genetic grounding to this process which has, thus far, been lacking. We chose to use the mutants of the knockout library instead of the products of a random mutagenesis as the knockouts allow an immediate identification of gene function in most cases.

Knockout strains displaying an altered morphological response to cold shock were classified according to the specific variation they displayed. Examples are shown in Figure 1. The “burst” phenotype was defined as displaying a large number of growing tips which stop growing, swell and then structurally fail leaving a pool of cytoplasm at the tip. The “fail” phenotype was defined as failing to display the apical branch phase characteristic of cold shock. In the “fail” response, growth proceeds normally with lateral branching following cold shock. The “thin” phenotype was defined by a very rapid decrease in hyphal diameter following cold shock. It was common to observe “thin” in combination with other altered cold shock responses. The “dense” phenotype was defined by displaying apical branching with visibly shorter distances between branch points following cold shock relative to the response in wild-type. The “weak” phenotype was defined as the opposite – an apical branch phase with visibly longer distances between branch points relative to wild-type following cold shock. Finally, the “cot-like” phenotype was characterized by a lack of apical branching, but a shift to tightly spaced lateral branches which morphologically resembled the growth of the traditional *cot* mutants at the restrictive temperature.

### Screen of *E. coli* cold shock gene homolog knockout set

A total of 68 *Neurospora* strains with knockouts of genes homologous to *E. coli* genes which alter transcription in response to cold shock (Phadtare and Inouye 2004) were screened. A total of 55 (81%) showed altered morphology to cold shock (Knockouts presenting alterations to the cold shock response are reported together in Table 1, sorted by phenotype). The knockouts displaying altered response to cold shock represent a variety of cellular functions. Phadtare and Inouye report genes which respond to cold shock by altering their transcription levels. Comparisons (χ^2^ not shown) between these transcription changes in *E. coli* and the cold shock phenotype displayed by these genes orthologs in *Neurospora* do not suggest there are any clear associations between transcription changes and cold shock morphology.

The screen of cold shock orthologs provides a test of the hypothesis that *E. coli* and *Neurospora* share a great deal of their cold shock response in common. The very high percentage of overlap between genes playing a role in these two widely separated organisms suggests that the two responses are functionally related.

### Screen of Morphological/Hyphal plate knockout sets

A total of 131 selected mutant strains from the *Neurospora* knockout library were previously segregated into two collections. The “Morphological” collection resulted in known morphological variations in the knockout strains. The “Hyphal” collection consisted of knockouts of genes previously suspected to play a role in hyphal growth. These two collections were screened for alterations to their response to cold shock. In total, 33 (25%) strains were identified (Table 2) that displayed variant cold shock responses. The altered responses fell into several phenotypic categories (Table 1).

The morphological/hyphal knockouts were previously screened for temperature-dependent branch density (Watters *et al.* 2011). Comparing the strains identified above with alterations to their cold shock response to those previously determined to show temperature-dependent branching we find only a modest overlap with the following strains showing altered phenotypes in both: NCU02333, NCU00830, NCU04242, NCU02114, NCU04264, and NCU03076. Examining the overlap statistically via χ^2^ (calculations not shown) yields a p value greater than 0.9, strongly suggesting that the overlap is random. This suggests that these two screens (cold shock *vs.* temperature sensitive branching during steady-state growth) are independent. This leads us to conclude that the cold shock response and temperature-dependent branching are independent aspects of cold adaptation, highlighting the different genes involved in short-term adaptation to the cold as opposed to those required for sustained growth in cold environments. Additional screens of the knockout library for strains displaying growth rate dependent branching, and comparing them to those with an altered cold shock response will allow us to further examine the apparent independence of these two morphological screens.

### Screen of transcription factor knockout set

A total of 145 *Neurospora* strains with knockouts in genes which function as transcription factors were screened for their response to cold shock. In all, 30 (20%) showed altered morphology to cold shock (Table 1).

As with the knockouts of orthologs of *E. coli* cold shock responding genes, the mutant strains identified in the additional screens show no observed correlations between the phenotypes observed and the annotated functions of the genes with a variety of functions being associated with the observed cold shock variations.

### Frequency of knockouts yielding alterations in cold shock was dependent on the category the the knockout

As detailed above, mutants screened represented three different categories of knockouts: *E. coli* cold-shock responding orthologs, *Neurospora* morphological/hyphal growth mutants, and *Neurospora* transcription factors. These three groups displayed altered cold shock responses at different rates with the majority (81%) of the *E. coli* orthologs showing altered responses and much lower frequencies (23% and 20% respectfully) of the morph/hyphal and transcription factor knockouts showing altered responses (Table 1). Additionally, the phenotypes of the altered cold shock response showed a non-random distribution with regard to the knockout set the mutant was associated with using χ^2^. Comparing knockout set *vs.* cold shock phenotype among those with alterations yields a χ^2^ of 32.2 and an associated p value < 1%. Much of the significance is coming from an over-representation of “dense” cold shock responses among otherwise unidentified (*i.e.*, “hypothetical protein”) transcription factors.

### Cold shock phenotype was not correlated to GO categorization of the knockouts

The cold shock phenotype of knockouts was compared to their gene ontology categorizations via χ^2^ analysis. Comparing cold shock phenotype to either its Molecular Function or Biological Process categorization failed to produce significant differences (p values of ∼0.75 and ∼0.5 respectfully). Thus, particular GO categorizations are not associated with specific altered phenotypes in the cold shock response.

The data were also examined to determine if there was a non-random association between knockouts which show any alteration to their cold shock response (regardless of the specific phenotype) and those that show the wild type response *vs.* their GO categorization. For both “Molecular Function” and “Biological Process” GO categories, no significant association was seen (via χ^2^, *P* = 0.4 and 0.5 respectfully), similarly failing to support the possibility that knockouts with specific GO categorizations are tied to the cold shock response.

### Cold shock phenotype was weakly associated With growth rate at 25° among transcription factor knockouts

Linear growth rates at 25° for the transcription factor knockouts reported by Carrillo *et al.* (2017) were compared via T-test for knockouts showing altered cold shock responses *vs.* those showing no alteration to the response. One possible association between growth rate at 25° and altered cold shock phenotype was found for the knockouts displaying a dense phenotype which showed statistically faster growth rates at 25° than those with no alterations to cold shock (T-test, *P* = 0.019). This is consistent with previous observations between growth rate and cold shock (Watters *et al.* 2000), however the opposite association (slow growth rates at 25° among mutants displaying weak cold shock responses or failure to respond) is not observed, as would be expected if growth rate was a key factor among the knockouts. Taken together, there appears to be, at best, a weak association between growth rate at 25° and alterations to the cold shock phenotype among the transcription factor knockout mutants. This stands in contrast to the observation in wild type *Neurospora* (Watters *et al.* 2000) that the morphology of the cold shock response was directly dependent on growth rate changes. This suggests that the altered morphologies observed among the knockout mutants are due to changes in gene activity associated with the knockouts and not simply the consequence of changes in growth rates in these mutants.

In conclusion, the gene functions highlighted by these screens (Table 1) are diverse. It is unclear how the diverse gene network, partially exposed here, coordinates for the function of temperature acclimatization. The results presented here demonstrate a strong relationship between the cold shock responses of *E. coli* and *Neurospora crassa*. The phenotype under examination here (morphological response to cold shock) appears to be influenced by a diverse network of genes. Similar diversity of function has been observed in other examinations of morphogenesis in *Neurospora* (Seiler & Plamann 2003). Further work on cold acclimatization should help clarify these connections.
